# Adolescent mental health problems in early stages of the COVID-19 pandemic were masked by lockdown measures and restrictions

**DOI:** 10.1192/bjo.2022.606

**Published:** 2022-11-23

**Authors:** Franziska Rockstroh, Michael Kaess

**Affiliations:** University Hospital of Child and Adolescent Psychiatry and Psychotherapy, University of Bern, Switzerland; University Hospital of Child and Adolescent Psychiatry and Psychotherapy, University of Bern, Switzerland; and Department of Child and Adolescent Psychiatry, Center for Psychosocial Medicine, University Hospital Heidelberg, Germany

**Keywords:** Self-harm, admissions, lockdown, COVID-19, paediatric mental illness

## Abstract

In the *BJPsych Open* Wong et al examined the influence of lockdown stringency during early stages of the COVID-19 pandemic on psychiatric emergency presentations among children and adolescents from ten countries. Data from March and April 2019 were compared with the same time frame in 2020, with particular focus on self-harm admissions. In this editorial, the publication is summarised and potential implications for the field and future studies are discussed.

In the past 2 years, the COVID-19 pandemic has had an impact on the lives of most of us and our children. To contain the virus and protect the health of citizens, many countries adopted lockdown measures varying in stringency. Adolescents were particularly affected by social distancing measures such as school closures and suspension of leisure activities. Although such changes may have had beneficial effects for some and stressors such as bullying and school pressure may have been reduced, feelings of loneliness due to social isolation, anxiety about the future and tensions at home could be observed as negative consequences.^1^ These phenomena are well-known stressors and risk factors for youth mental health problems, and self-harm in particular. Self-harm is widely considered as a transdiagnostic marker for psychopathology^2^ and can reflect a manifestation of severe emotion dysregulation. Even before the pandemic, psychiatric emergency presentations among children and adolescents have increased noticeably over recent decades,^3^ with a rise also in self-harm referral rates.^4^ At the same time, help-seeking behaviour among adolescents who self-harm is generally low.^5^ Barriers for seeking help can include fear of stigmatisation, insufficient psychoeducation and lack of adequate treatment offers. During a pandemic, the fear of contagion, particularly in a hospital environment, may hinder professional help-seeking even further.

The influence of COVID-19 on child and adolescent mental health seems to be complex. Regardless of negative effects the pandemic may have had, the number of psychiatric emergency admissions was shown to deviate from the long-standing upwards trajectory. In a large retrospective cohort study, presentations were found to have decreased significantly in March and April 2020 compared with the same period in 2019, during which no lockdown measures were in place.^6^ Among all admissions, rates of self-harm increased, though. Based on these data, Wong and colleagues conducted further analyses on the effect of lockdown stringency, which varied between sites, on paediatric self-harm presentations during COVID-19,^7^ which are discussed in the present editorial.

## Wong et al's paper: outline and results

Fourteen sites were aggregated from 23 emergency departments in 10 countries according to geographical and sociodemographic characteristics. A total of *n* = 2073 presentations were analysed. The 10 countries were: Austria, England, Hungary, Ireland, Italy, Oman, Scotland, Serbia, Turkey and United Arab Emirates. Variables of interest included socioeconomic deprivation and lockdown stringency at site level and for each presentation, self-harm (yes/no) and psychiatric diagnoses. If the reason for admission was self-harm, the behaviour was further characterised according to severity, method, intent and self-harm history, and up to three precipitants to the incident could be defined. The aim of the study was to further disentangle the mechanisms involved in psychiatric emergency presentations for self-harm during early stages of the COVID-19 pandemic, with a particular focus on the effects of lockdown measure stringency on temporal change in admission numbers and self-harm characteristics. Specifically, the authors analysed whether the stringency of lockdown measures was a mediator of year on (a) number of presentations in general, (b) self-harm presentations and (c) ratio of self-harm presentation to all presentations.

The mediation effect was confirmed: higher lockdown stringency was linked to lower numbers of both psychiatric emergency and self-harm presentations, and when controlling for stringency the previously detected reduction in general and self-harm admissions from 2019 to 2020 was no longer significant. The lower number of emergency psychiatric presentations in 2020 compared with 2019 seems to be a result of lockdown stringency and not an indicator of actual improvement of mental health problems among adolescents. According to these data, the upward trajectory of admissions observed in previous years would have most likely continued, had there been no restricting measures in place. Furthermore, the more stringent the lockdown measures were, the higher the ratio of self-harm presentations to all emergency psychiatric presentations was. Among self-harm presentations, additional analyses revealed further effects of stringency on self-harm. Stringency did not have an influence on any self-harm characteristics, such as severity, suicidal intent or numbers of psychiatric diagnoses. Three-quarters of patients with self-harm were female, but the ratio of male and looked after adolescents presenting with self-harm was significantly increased in areas with a higher stringency index compared with less strict regions and the commonly observed gap between genders^3^ was reduced. As could be expected, school pressure and problems with friends as precipitants of self-harm were less likely in areas with more stringent lockdown measures, whereas self-harm due to social isolation was more frequently reported.

## Discussion

In this paper, many important questions about self-harm, help-seeking behaviour and effects of lockdown measures on children and adolescents were addressed. Even though consequences of social distancing on people's mental health have been the focus of many research groups worldwide since pandemic-related measures were first implemented, comprehensive and meaningful data are still limited. One big strength of the current study was the inclusion of data from high- and low-income countries with diverse mental health systems and a variety of political reactions to the COVID-19 pandemic. By dismantling the measures implemented over different sites and including a stringency index, the impact of various levels of lockdown could be investigated in an international retrospective cohort.

The stringency of measures had a strong influence on the reduction in psychiatric and self-harm presentations, and the stricter the area, the higher the proportion of self-harm presentations. This impressively demonstrates that fewer presentations at psychiatric emergency units are not necessarily representative of actual reductions in self-harm behaviour and psychological distress in general and raises the question of whether admissions to psychiatric emergency units are valid markers for public mental health. Barriers to seeking help were potentially increased even more due to the pandemic and crises may have simply not been detected. Increases in psychological stress during the pandemic have been confirmed among children and adolescents^1,8^ and the gap between mental health problems and help-seeking behaviour may have widened even further, at least during times of lockdown. It should be noted that presentation numbers among males and children and adolescents who were looked after by a local authority increased with stringency. It remains unclear whether they were particularly affected by lockdown measures during this trying time or whether mental health problems were more frequently detected because of time spent with family and other caregivers. Help-seeking behaviour is particularly low in these populations and efforts to increase mental health support need to target such vulnerable groups.

Owing to the limited time frame examined in the paper, no statements can be made regarding admission numbers at other stages of the COVID-19 pandemic. It will be interesting to see future analyses on general psychiatric presentation and self-harm patterns over longer periods of the pandemic, particularly after many strict lockdown measures were lifted. Indeed, it is possible that lower help-seeking opportunities during lockdown may have affected presentations to both emergency units and the overall mental healthcare system after periods of strict lockdown, which would fit the overall picture of increased service use in many mental healthcare systems worldwide during the pandemic and would be expected, given the emerging evidence for a deterioration in youth mental health.^8^ Such data will contribute important knowledge regarding long-term self-harm trajectories and mental health trends during a global crisis. The urgent need for low-threshold support has been discussed in detail in the field and such sophisticated analyses provide a meaningful foundation for further research and critical policy implications.

## Data Availability

Data availability is not applicable to this article as no new data were created or analysed in this study.

## References

[1] Panchal U, Salazar de Pablo G, Franco M, Moreno C, Parellada M, Arango C, The impact of COVID-19 lockdown on child and adolescent mental health: systematic review. Eur Child Adolesc Psychiatry [Epub ahead of print] 18 Aug 2021. Available from: 10.1007/s00787-021-01856-w. PMC8371430 34406494

[2] Ghinea D, Edinger A, Parzer P, Koenig J, Resch F, Kaess M. Non-suicidal self-injury disorder as a stand-alone diagnosis in a consecutive help-seeking sample of adolescents. J Affect Disord 2020; 274: 1122–5. 32663940 10.1016/j.jad.2020.06.009

[3] McManus S, Gunnell D, Cooper C, Bebbington PE, Howard LM, Brugha T, Prevalence of non-suicidal self-harm and service contact in England, 2000–14: repeated cross-sectional surveys of the general population. Lancet Psychiatry 2019; 6: 573–81. 31175059 10.1016/S2215-0366(19)30188-9 PMC7646286

[4] Burstein B, Agostino H, Greenfield B. Suicidal attempts and ideation among children and adolescents in US emergency departments, 2007–2015. JAMA Pediatrics 2019; 173: 598–600. 30958529 10.1001/jamapediatrics.2019.0464 PMC6547071

[5] Rowe SL, French RS, Henderson C, Ougrin D, Slade M, Moran P. Help-seeking behaviour and adolescent self-harm: a systematic review. Aust N Z J Psychiatry 2014; 48: 1083–95. 25335872 10.1177/0004867414555718

[6] Ougrin D, Wong BHC, Vaezinejad M, Plener PL, Mehdi T, Romaniuk L, Pandemic-related emergency psychiatric presentations for self-harm of children and adolescents in 10 countries (PREP-kids): a retrospective international cohort study. Eur Child Adolesc Psychiatry 2022; 31(7): 1–13. 10.1007/s00787-021-01741-6 PMC7937052 33677628

[7] Wong BHC, Vaezinejad M, Plener PL, Mehdi T, Romaniuk L, Barrett E, Lockdown stringency and paediatric self-harm presentations during COVID-19 pandemic: retrospective cohort study. BJPsych Open 2022; 8(2): e75. 35322782 10.1192/bjo.2022.41 PMC8963968

[8] Racine N, McArthur BA, Cooke JE, Eirich R, Zhu J, Madigan S. Global prevalence of depressive and anxiety symptoms in children and adolescents during COVID-19: a meta-analysis. JAMA Pediatrics 2021; 175: 1142–50. 34369987 10.1001/jamapediatrics.2021.2482 PMC8353576

